# Human-based approaches to pharmacology and cardiology: an interdisciplinary and intersectorial workshop

**DOI:** 10.1093/europace/euv320

**Published:** 2015-11-29

**Authors:** Blanca Rodriguez, Annamaria Carusi, Najah Abi-Gerges, Rina Ariga, Oliver Britton, Gil Bub, Alfonso Bueno-Orovio, Rebecca A.B. Burton, Valentina Carapella, Louie Cardone-Noott, Matthew J. Daniels, Mark R. Davies, Sara Dutta, Andre Ghetti, Vicente Grau, Stephen Harmer, Ivan Kopljar, Pier Lambiase, Hua Rong Lu, Aurore Lyon, Ana Minchole, Anna Muszkiewicz, Julien Oster, Michelangelo Paci, Elisa Passini, Stefano Severi, Peter Taggart, Andy Tinker, Jean-Pierre Valentin, Andras Varro, Mikael Wallman, Xin Zhou

**Affiliations:** 1 Department of Computer Science, University of Oxford, Oxford, UK; 2 Medical Humanities, University of Sheffield, Sheffield, UK; 3 AnaBios Corporation, San Diego Science Center, San Diego, CA 92109, USA; 4 Radcliffe Department of Medicine, University of Oxford, John Radcliffe Hospital, Oxford, UK; 5 Department of Physiology, Anatomy and Genetics, University of Oxford, Oxford, UK; 6 Department of Engineering Science, University of Oxford, Oxford, UK; 7 QT-Informatics Limited, Macclesfield, UK; 8 William Harvey Heart Centre, Barts and The London School of Medicine and Dentistry, Queen Mary University of London, Charterhouse Square, London, UK; 9 Discovery Sciences, Dis&Dev Research, Janssen Pharmaceutical NV, Beerse, Belgium; 10 Institute of Cardiovascular Science, University College London, Bars Heart Centre, London, UK; 11 Department of Electronics and Communications Engineering, Tampere University of Technology, BioMediTech, Tampere, Finland; 12 Department of Electrical, Electronic and Information Engineering, University of Bologna, Cesena 47521, Italy; 13 Non-Clinical Development, UCB-Biopharma, Braine L'Alleud B-1420, Belgium; 14 University of Szeged, Szeged, Hungary; 15 Fraunhofer-Chambers Centre, Gothenburg, Sweden

**Keywords:** Human-based methods, Computational approaches, Human electrophysiology, Stem-cell-derived cardiomyocytes, Computer modelling and simulations, Arrhythmias, Biomarkers

## Abstract

Both biomedical research and clinical practice rely on complex datasets for the physiological and genetic characterization of human hearts in health and disease. Given the complexity and variety of approaches and recordings, there is now growing recognition of the need to embed computational methods in cardiovascular medicine and science for analysis, integration and prediction. This paper describes a Workshop on Computational Cardiovascular Science that created an international, interdisciplinary and inter-sectorial forum to define the next steps for a human-based approach to disease supported by computational methodologies. The main ideas highlighted were (i) a shift towards human-based methodologies, spurred by advances in new *in silico*, *in vivo*, *in vitro*, and *ex vivo* techniques and the increasing acknowledgement of the limitations of animal models. (ii) Computational approaches complement, expand, bridge, and integrate *in vitro*, *in vivo*, and *ex vivo* experimental and clinical data and methods, and as such they are an integral part of human-based methodologies in pharmacology and medicine. (iii) The effective implementation of multi- and interdisciplinary approaches, teams, and training combining and integrating computational methods with experimental and clinical approaches across academia, industry, and healthcare settings is a priority. (iv) The human-based cross-disciplinary approach requires experts in specific methodologies and domains, who also have the capacity to communicate and collaborate across disciplines and cross-sector environments. (v) This new translational domain for human-based cardiology and pharmacology requires new partnerships supported financially and institutionally across sectors. Institutional, organizational, and social barriers must be identified, understood and overcome in each specific setting.

## Motivation

Both biomedical research and clinical practice rely on complex datasets for the physiological and genetic characterization of human hearts in health and disease. The information shaping our knowledge of human hearts is obtained from a variety of techniques and models, including recordings obtained *in vivo* invasively and non-invasively, in *ex vivo* tissue and isolated human adult cardiomyocytes recordings, and more recently *in vitro* using human stem-cell-derived cardiomyocytes. Increasing evidence suggests that non-human animal models may have limited ability to predict human *in vivo* effects due to important species differences between humans, dogs, guinea pigs, and rabbits.^1–3^ Thus, methods firmly rooted in understanding physiology and pathophysiology in humans are clearly needed.

Advances in imaging technologies such as the multiple modalities of cardiac magnetic resonance (CMR) are combined with body surface or intra-cardiac electrophysiological recordings to evaluate in specific patients the *in vivo* structural and functional implications of cardiac disease. Recent progress in research using human cardiomyocytes derived from induced pluripotent stem cells promises exciting new developments as it allows the *in vitro* characterization of the phenotype of cardiomyocytes of specific patients, and therefore has the potential of introducing the flexibility of *in vitro* methodologies in personalized medicine. Furthermore, *ex vivo* tissue from biopsies or from donor human hearts provides measurements of tissue, cellular, and ionic properties of human adult cardiomyocytes in non-diseased and diseased conditions.

Each of these types of human-based assays and measurements provides us a single snapshot from one perspective of a complex set of variables through both time and spatial dimensions. In turn, this complex set of variables is able to define and explain the myriad of dynamic mechanisms and properties that underlie the activity of human hearts in health and disease. Given the complexity and variety of approaches and recordings, there is now growing recognition of the need to embed computational methods in cardiovascular medicine and science for analysis, integration, and prediction. Computational approaches in biomedicine range over a variety of techniques for signal, data, and image analysis, but also importantly multiscale modelling and simulation. Together, they provide a synergistic approach to organize and augment the information obtained from experimental and clinical recordings. The benefits gained include the quantitative analysis and organized reassembly of multiscale and multimodality datasets to probe, challenge and expand our knowledge of the complex and dynamic interactions in cardiac electrophysiology. Advances in computational cardiac electrophysiology were recently illustrated in two dedicated special issues of *Europace 2014*.^4,5^ Furthermore, new initiatives such as the Comprehensive *in vitro* Proarrhythmia Assay (CiPA) launched by the United States Food and Drug Administration (FDA) recognize the potential of human-based *in silico* and *in vitro* approaches as a new paradigm for drug safety assessment.^6^

**Figure 1:**
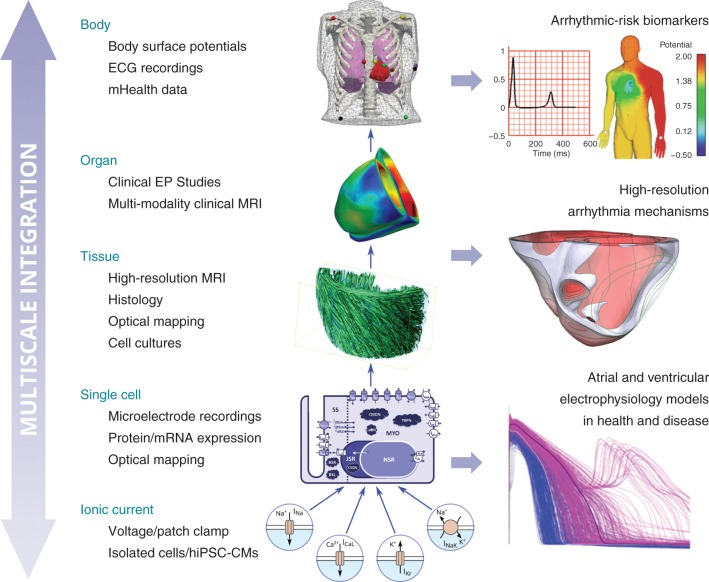
Sources of experimental data integrated in computational models of human cardiac electrophysiology, and applications in physiology. Ionic current models are constructed mostly based on voltage/patch clamp data from *ex vivo* and *in vitro* preparations. The integration of ionic current models in single cell models, accounting for variability in protein expression and disease remodelling, allows for the simulation of the action potential and electrolyte concentrations in healthy and disease. Additionally, cardiac simulations at the whole organ and body levels require the construction of image-based anatomical models. When coupled to mathematical descriptions of electrical excitation through cardiac tissue, they allow for the high-resolution investigation of arrhythmia mechanisms based on clinical electrophysiology studies, as for the interpretation and identification of arrhythmic-risk biomarkers at the surface potential level. Transmural visualization of ventricular myofibre orientation, adapted from reference 8 with permission. ECG/whole body simulation, adapted from reference 9 with permission.

On 17 September 2014, a Workshop on Computational Cardiovascular Science was hosted at the University of Oxford with the aim of creating an international, interdisciplinary, and inter-sectorial forum to discuss current trends in computational technologies to augment cardiovascular physiology, pharmacology, and medicine, and to propose solutions for the replacement, refinement and reduction of animal experimentation.^7^ Invited participants included experts in cardiology, computer science, physiology, pharmacology, philosophy, and biomedical engineering from academia and industry, from 11 Universities and 12 companies, from UK, several countries in Europe, USA, and Japan. In this paper, we aim at describing the main ideas discussed during the meeting, rather than providing a thorough review of the literature. *Table 1* summarizes the forms of human-based *in vivo*, *ex vivo*, *in vitro*, and *in silico* experiments and techniques discussed, which are further illustrated in *Figure 1*.

**Table 1:** Summary of human-based *in vivo*, *ex vivo,* and *in vitro* techniques and *in silico* approaches

Human-based	Data acquisition technique	Limitations of the data	Progress of *in silico* approaches
*In vivo*	Electrophysiological recordings during clinical procedures [Taggart, Zhou]	– Limited data sets due to ethical and practical obstacles; – Datasets derived from diseased hearts; – Inter-patient variability; – Limited experimentation	– Signal analysis and integration; – Multiscale electrophysiological models and simulations; – Population of models to mimic action potential variability
	Multimodality imaging including magnetic resonance [Ariga, Grau]	– Limited data sets; – No experimentation	– Image analysis (tissue characterization); – Ventricular shape analysis; – Structural models and simulations – Computer models for link between structure and diffusion; – And link between micro structure and function
	Body surface potentials, electrocardiogram [Minchole, Lu]	– Captures global patterns of heart behaviour; – Variability	– Automated quantification of ECG features for clinical diagnosis and identification of new bioamarkers (morphological QRS or T-wave based, iCEB); – Electrocardiographic imaging; – Multiscale human heart simulations from ion channel and microstructure to the electrocardiogram
	mHealth recordings through mobile devices [Oster]	– Very large quantities of data not amenable to manual analysis; – Noisy and patchy data; – Social, ethical and legal challenges	– Automated and semi-automated techniques for analysis, such as machine learning, implementable on mobile phones
	Isolated human primary cells and non-clinical, real-world data from biopsies and medical histories [Ghetti]	– Limited data sets; – Social, ethical and legal challenges	– Computational models to integrate experimental data and to investigate multiscale mechanisms of disease and pharmacological interventions
*Ex vivo*	Microelectrode, optical mapping, patch clamp, protein, and mRNA expression [Varro, Britton, Dutta]	– Limited availability, and mostly from diseased hearts; – Variability; – Difficulty of technique implementation (cell isolation; current separation); – Change of properties due to cell isolation	– Data analysis and integration; – Multiscale models for greater contextualization; – Investigation of variability through approaches such as population of models
*In vitro*	Human cardiomyocytes derived from induced pluripotent stem cells (hiPSC-CMs) [Daniels, Severi, Kopljar, Harmer]	– Inconsistent immaturity; – Variability and associated difficulty of comparison	– Models to investigate variability, assist interpretation, and facilitate translation to *in vivo* cells – Models to investigate gene mutations; – Models for drug safety assessment
	Cell cultures and high-speed optical imaging [Burton]	– Limited cross-institution and cross-sector access to experiments	– Multiscale modelling to explain dynamics in heterogeneous preparations

The main ideas highlighted through the workshop were the following:

- A shift towards human-based methodologies in pharmacology and medicine is occurring, spurred by advances in new *in silico*, *in vivo*, *in vitro,* and *ex vivo* techniques and the increasing acknowledgement of the limitations of animal models.
- Computational approaches complement, expand, bridge, and integrate *in vitro*, *in vivo,* and *ex vivo* experimental and clinical data and methods, and as such they are an integral part of human-based methodologies in pharmacology and medicine.
- The effective implementation of multi- and interdisciplinary approaches, teams, and training combining and integrating computational methods with experimental and clinical approaches across academia, industry, and healthcare settings is a priority.
- The human-based cross-disciplinary approach requires experts in specific methodologies and domains, who also have the capacity to communicate and collaborate across disciplines and to work productively in interdisciplinary and cross-sector environments.
- This new translational domain for human-based cardiology and pharmacology requires new partnerships supported financially and institutionally across pharma and biotech industry, contract research organizations (CRO), academia and research institutes, technology and service providers, non-profit and governmental organizations, and regulatory agencies. Institutional, organizational, and social barriers must be identified, understood, and overcome in each specific setting.

## Description of the workshop

The workshop consisted of four sessions aiming at exploring different aspects of human-based cardiovascular science, and specifically the synergies with *in silico* approaches in the three main experimental settings, i.e. *in vivo*, *in vitro*, and *ex vivo. Table 1* summarizes the forms of experiments and techniques discussed.

### Human *in vivo* cardiovascular science

Prof. Peter Taggart examined the challenges involved in obtaining basic electrophysiological data from *in vivo* human subjects, during routine clinical procedures. The example discussed was the application of percutaneous transluminal coronary angioplasty (PTCA) to study *in vivo* the effect of myocardial ischaemia (a major cause of mortality) on human electrophysiology, by recording either monophasic action potentials or unipolar electrograms on the ventricular endocardium in the region served by the artery undergoing PTCA. *In vivo* electrophysiological recording techniques include the use of the multi-electrode sock (264 electrodes) over the human ventricles to study global patterns of activation such as ventricular fibrillation, action potential duration (APD) changes, and post-repolarization refractoriness during early ischaemia and the demonstration of mechano-electric feedback in humans.^10–20^ Findings addressed the debate as to whether rotors or multiple wavelets drive ventricular fibrillation (VF) and showed that both coexist in human VF, and the very rapid time course of the early electrophysiological changes during early ischaemia in humans. *In vivo* electrophysiological recordings are critical for translational research (from basic to clinical) but they are limited due to considerations for patient comfort and safety. Therefore, they need to be complemented by and combined with alternative ways of probing the human heart, including non-invasive *in vivo* imaging as well as *ex vivo* investigations, as described below.

Dr Rina Ariga provided an overview of multimodality magnetic resonance imaging and its application in patients with hypertrophic cardiomyopathy (HCM). HCM is the most common genetic heart disease (affects 1 in 500)^21^ and the commonest cause of sudden cardiac death in the young.^22^ Transthoracic echocardiography (TTE) is routinely used to assess HCM, but is limited in patients with poor acoustic windows or poor visualization of some LV regions. CMR is now the gold standard in assessing mass, hypertrophy, volume, and function in HCM due to high spatial and temporal resolution.^23^ Unlike TTE, CMR also provides tissue characterization using late gadolinium enhancement (detects focal fibrosis which has been associated with ventricular arrhythmias and SCD)^24^ and T1 mapping (detects diffuse and focal fibrosis). CMR also offers insights into several hallmark features of HCM that are potential contributors of disease progression using novel techniques such as stress perfusion imaging (impaired perfusion),^25^ blood oxygen level dependent (BOLD) imaging at stress (deoxygenation at stress),^26^ phosphorus MR spectroscopy (abnormal myocardial energetics at rest with further deficit in exercise),^27^ and most recently, diffusion tensor imaging (to assess fibre disarray).^28^ CMR is not only a useful imaging adjunct in cases with limited TTE views, but also provides accurate disease characterization of subtle differences. Research using CMR is improving our understanding of this complex heterogeneous disease and is helping to guide risk stratification and treatment strategies.^29^

Prof. Vicente Grau spoke about methods to investigate myocardial microstructure, including quantification using imaging as well as determination of functional repercussions of microstructural changes, investigated using computational modelling and simulation. Recent developments in MRI technology, in particular using diffusion sensitive sequences, allow the quantification of microstructure, initially in fixed hearts^8,30,31^ or in hearts at different stages of contraction.^32^ Diffusion MRI uses an indirect measurement to estimate cardiac structure, and its relationship to cardiac microstructure is not fully understood. Two methods to improve this understanding were discussed. Histology offers direct insights into microstructure, but three-dimensional reconstruction from histological slices is challenging.^33^ Computational models, simulating water diffusion and MRI acquisition sequences, can be used to provide a direct link between structure and diffusion.^34^ The relationship between microstructural and functional changes is not fully understood, and here computational models can again provide a unique tool as shown for example in references.^35–37^

**Figure 2:**
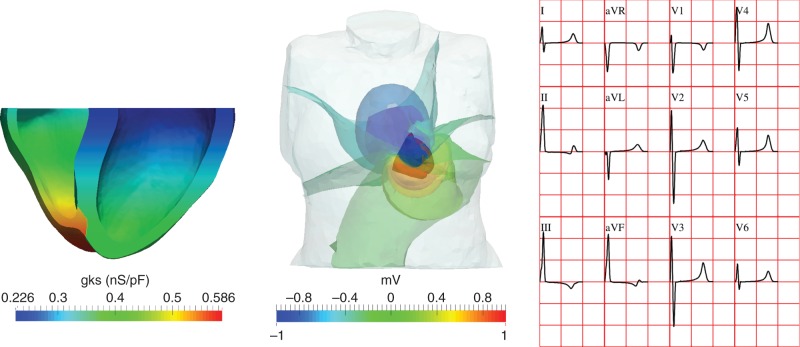
Computer simulation of the human heart electrophysiology from ion channel to body surface potentials and the electrocardiogram. Simulations are conducted using human biophysically detailed models considering heterogeneity in specific ionic properties (left, colour scale correspond to the maximum conductance of the slow component of the delayed rectifying current) to determine their effect on the spatiotemporal evolution of electrical potentials across the whole torso (middle, extracellular potentials throughout the torso) and on the ECG (right, main leads displayed).

In addition to imaging, clinical *in vivo* recordings to evaluate the human heart include the surface electrocardiograms (ECGs), a rapid and cost-effective method to acquire non-invasive recordings. A large body of research has been devoted to the quantification of ECG features for patient stratification in terms of arrhythmic risk and disease.^38^ Dr Ana Mincholé described computational approaches applied to the ECG for the detection of electrophysiological abnormalities and patient stratification in HCM. Using a database of Holter recordings obtained in HCM patients and volunteers, she described the quantification of standard ECG-based biomarkers such as QT T peak to T end, as well as new mathematical model-based morphological features of the QRS complex and T wave morphology.^39,40^ Dr Minchole also described how information about the electrophysiological and structural signature of disease from the ionic to the whole organ level can be integrated in multiscale human heart models and used in the development and understanding of ECG-based biomarkers.^41^ Multiscale simulations as illustrated in *Figure 2* allow the identification of key structural and functional factors that determine each of the ECG biomarkers and provide a deeper and more precise understanding of the information each of the biomarkers conveys.^42^ The new knowledge also aids in the identification of more selective and specific biomarkers for specific disease conditions with complex functional and structural signatures such as HCM or myocardial infarction. Body surface ECGs combined with imaging data using mathematical algorithms to non-invasively reconstruct the electrical activity on the epicardial surface of the heart *in vivo* as demonstrated for example in references.^43–45^

### Human *ex vivo* and *in vitro* cardiovascular science

**Figure 3:**
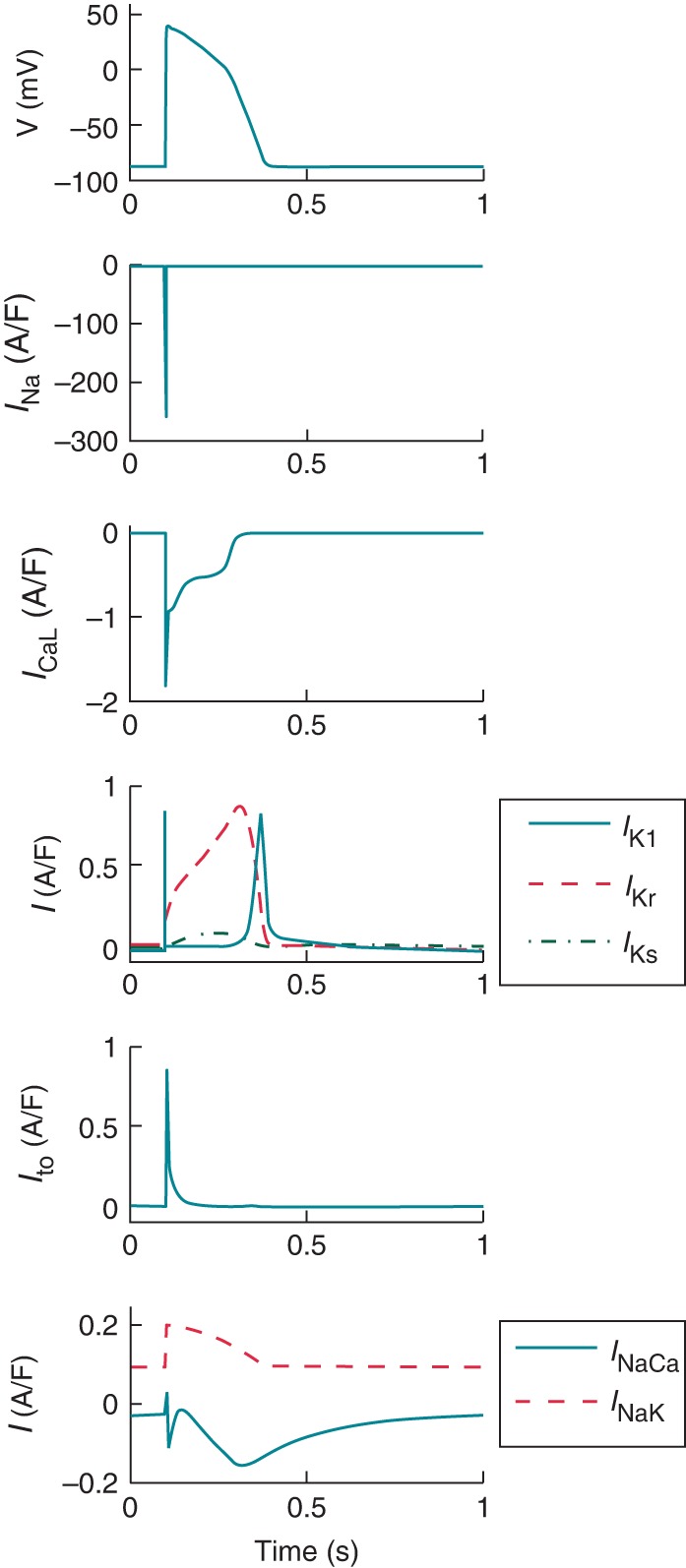
Simulation of the human ventricular action potential and the underlying ionic currents. From top to bottom, time course of the action potential, sodium current (*I*_Na_), L-type calcium current, the rapid and slow component of the delayed rectifying current (*I*_Kr_, *I*_Ks_) and the inward rectifying current (*I*_K1_), the transient outward current (*I*_to_) and the sodium potassium pump (*I*_NaK_) and the sodium calcium exchanger (*I*_NaCa_).

Prof. Andras Varro began by discussing the knowledge we have of human electrophysiology largely through classic techniques such as conventional microelectrode and more modern patch clamp techniques, protein and RNA expression approaches using human cardiomyocytes from biopsies or donor hearts.^46–48^ Obtaining good representative experimental human data is restricted by a number of practical problems. Sources of tissue particularly ventricular cells are difficult to obtain and isolation of cells is complicated by disease. The source of undiseased donor hearts is particularly limited. The process of cell isolation may also lead to changes from properties studied in multicellular tissue preparations. Furthermore, the separation of each of the ionic currents illustrated in *Figure 3* is problematic since the available pharmacological inhibitors are not totally selective. Finally, Prof. Varro highlighted the importance of human-based studies rather than highlighted the use of animal models. It is often underappreciated that there are substantial species differences between human and even large mammals, such as the dog, considered to be a representative model.^49,50^ For example, marked differences in ventricular repolarization reserve have been reported between human and dog,^49^ with larger rapid component of the delayed rectifying current (*I*_Kr_) in human but stronger slow component (*I*_Ks_) and inward rectifying current (*I*_K1_) in the dog. Consequently, APD prolongation caused by selective *I*_Kr_ block is three-fold larger in human than in dog, which suggests caution in translation of animal findings to human. Species-specific differences in pharmacological action between animal models have been also described,^50^ with rabbit exhibiting larger APD prolongation and proneness to repolarization abnormalities upon selective *I*_Kr_ block compared to others. Computational modelling and simulation can facilitate interspecies comparison by identifying and addressing differences and maximizing the re-use of experimental data from human hearts.

Dr Oliver Britton described how the complex electrophysiological datasets obtained from *ex vivo* human hearts have been integrated in multiscale computer models, specifically focusing on human data over the past decade^51–54^ as examples. He described the recent construction of a population of human ventricular cell computational models that captures the inter-subject variability seen in action potential recordings from human ventricular tissue preparations from Prof Varro's laboratory. The models in the population have a wide range of different configurations of ionic current strengths to mimic variability in a population as in reference 55. The team investigated whether there were configurations that were particularly vulnerable to developing repolarization abnormalities such as alternans and early afterdepolarizations (EADs), in response to blockade of different combinations of currents known to be important in repolarization—rapid delayed rectifying (*I*_Kr_), slow delayed rectifying (*I*_Ks_), inward rectifying (*I*_K1_), and late calcium (*I*_CaL_) current. The computational approach therefore integrates and extends experimental recordings, generating predictions and refining hypotheses about proarrhythmic mechanisms that can then be tested experimentally. Importantly, the *in silico* human models provide a multiscale framework to investigate with high spatiotemporal resolution key ionic mechanisms in drug safety and efficacy in human, with high degree of flexibility in the possible interventions (such as heart rate, concentrations, and adrenergic challenge) compared to experiments.

Prof. Matt Daniels reviewed the status of human cardiomyocytes derived from induced pluripotent stem cells (hiPSC-CMs). He saw three uses for these cells:

- screening for cardiotoxicity of novel therapeutics,
- modelling and drug discovery for inherited cardiac conditions and
- in the medium to long term, regenerative cell therapy.

**Figure 4:**
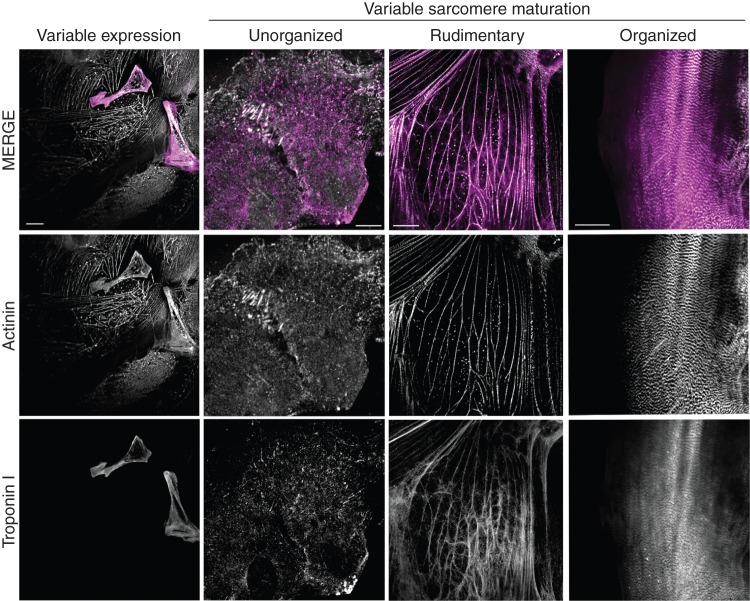
Stem-cell-derived cardiomyocytes have variable phenotypes: current methods of stem cell differentiation produce mixed populations at two distinct levels—gene expression, and post-transcription. This is demonstrated for sarcomeric morphology here, with the panel on the left showing two cells in the field of view positive for the z-disc marker alpha-actinin (white), and the thin filament protein troponin I (magenta). However, further heterogeneity exists even within the cell populations expressing both markers, as only some cells demonstrate ordered sarcomeric units with clear cell polarity (panel on the right). Methods to eliminate (or compensate for) this will be needed to enable small differences between samples to be identified above the noise of the difference within the sample. Scale bar 10 µm. The human ES line OXF2 was grown to confluency on Matrigel and differentiated as described in reference 59. Cells were dissociated by incubation with trypsin/EDTA (0.05%, Lifetech) for 15 min at room temperature prior to seeding onto 0.1% gelatin coated glass coverslips. Ten days after seeding, cells were fixed in 4% PFA (10 min, room temperature), permeabilized (0.1% Triton X-100 in Tris-buffered saline), and blocked with 2% BSA plus 0.001% sodium azide in TBS-T (1 h RT) and incubated with Primary antibodies (mouse monoclonal anti alpha-actinin, (sigma), and rabbit polyclonal anti-troponin T, prior to washing and incubation with Fab fragment anti-mouse 488, and anti-rabbit 568 (molecular probes). Images were acquired on an upright Leica SP5 confocal with a 63× lens.

While there is progress in scaling up production of these cells,^56^ functional maturity is still a major impediment. Stem cell derivatives in many ways (force generation, gene expression, sarcomeric maturity, electrophysiological properties, etc.) resemble immature neonatal cardiac substrates.^57^ However, there is one key difference that is rather unique to these cells and best illustrated by comparison to the existing alternatives. Adult cardiomyocytes are consistently mature; neonatal cardiomyocytes are consistently immature. By contrast, stem cell derivatives display an inconsistent immaturity (*Figure 4*) such that in any experimental test to date, variability within the sample is typically one order of magnitude of measures such as APD and cycle length as shown in reference 58. This will complicate comparisons between samples, which are typically made on small numbers of cells.

Dr Stefano Severi showed how a computational approach can be supportive and complementary to the functional *in vitro* study of hiPSC-CMs. In exploiting hiPSC-CMs as *in vitro* models for the electrophysiological effects of evolving or new drugs, a detailed understanding of the electrophysiological properties of hiPSC-CMs is necessary. *In silico* models of hiPSC-CMs action potential have been constructed for control^60^ and some genetic mutations, such as those causing long QT (LQT) syndrome type 1 (LQT1),^61^ type 2, and type 3,^62^ based on recent electrophysiological measurements^63–66^ and validated against drug administration. Computer simulations showed that in principle hiPSC-CMs are qualitatively consistent with adult CMs in response to many current blockers,^67^ but differences also emerged. Moreover, hiPSC-CMs show a highly variable electrophysiological behaviour, namely a variable and depolarized resting potential and diverse rates of spontaneous action potentials. Such high variability can be perceived as a limiting factor to the application of the computational approach to hiPSC-CMs. Indeed, computational models of cell electrophysiology are usually developed on the basis of the average values of quantities measured in *in vitro* experiments. The underlying idea is that the predictions obtained with the model of the ‘average cell’ can apply to all the cells with a tolerance that is of the same order of the variability of the data on which the model is based. Therefore, high variability in the data results in predictions with lower reliability. One way to overcome this limitation is to include the variability within the model itself (which is no longer a model of the ‘average cell’) in order to take it into account both in the investigation of physiological mechanisms and in model-based predictions. The aforementioned population of models approach is a relevant and promising example. Furthermore, when experimental data show high variability, computational models can help to identify the causes of such heterogeneity and potentially help to reduce it. As a relevant example, computational analysis can be used to assess to what extent the variability is due to (i) different experimental systems (e.g. cell lines) that could then be described by different, specific, computational models (ii) differences in the expression of ionic channels from cell to cell, which is eventually much larger than in adult cardiomyocytes or (iii) lack of robustness in the electrical activity of incompletely mature hiPSC-CMs. An example of the latter case are the effects of small changes in depolarizing currents, which can lead to dramatic changes in the rate of spontaneous beating in hiPSC-CMs, whereas they lead to only minor changes in resting potential in adult cardiomyocytes. In this sense, the variability can be reduced if observed in the model parameter's space since cells showing very different APs could be quite close in terms of their ionic current maximal conductances.

Dr Ivan Kopljar continued the theme looking at experimental studies on hiPS-CMs in safety assessments in the drug development setting, with an emphasis on the investigation of long-term drug effects. Currently, various technologies such as multi-electrode array (MEA), Ca^2+^ transient, impedance, and optical action potential measurements are applied on hiPSC-CMs for their characterization. Their potential has been highlighted by the CiPA initiative launched by the FDA. On the other hand, drug-induced delayed and chronic cardiotoxicity is one of the main risk for drug withdrawal from the market. Therefore, Dr Kopljar described investigations of the acute and delayed (5 days) effects of various oncological compounds on hiPS-CMs using an impedance-based functional assay. Different functional parameters such as beat rate, cell index and incidence of arrhythmia-like events were evaluated, and indicate that the hiPS-CMs can be used to detect different levels of acute and chronic cardiotoxicity and could be valuable in drug safety.

In another industrial perspective, Dr Najah Abi-Gerges discussed currently employed strategies in the pharmaceutical industry for cardiotoxicity screening. He gave an overview of the issue pointing out the high attrition rate of new molecules during the iterative drug discovery process: a significant number of these are caused by cardiotoxicity.^68^ He highlighted the extent to which some safe new drugs are not developed because of these potential concerns. The new CiPA initiative attempts to address this issue.^6^ CiPA proposes a paradigm based on screening new molecules against specific cardiac ion channels combined with integrative computer modelling to predict the proarrhythmic potential of new drugs. This is subsequently combined with non-rodent and early human clinical studies to assess drug effects on QTc measurements. Dr Abi-Gerges highlighted the need for further developing robust and predictive *in silico* models that represent native myocyte physiology and the heart of healthy volunteers and patients. Such models will predict acute and chronic drug effects with high predictive value on ECG abnormalities other than QT/proarrhythmia, heart rate, contractility, blood pressure, and cardiac structure. Dr Abi-Gerges concluded by advocating that scientists, modellers, regulators, and pharmaceutical industry are currently well positioned to shape how future cardiac modelling would positively impact drug development.

### Arrhythmia mechanisms and biomarkers

A fourth session presented the use of experimental and computational methods for investigations into arrhythmia mechanisms and biomarkers. Dr Stephen Harmer continued the theme of iPSC technologies, in this case for investigations on mechanisms of disease pathogenesis in LQT1 using iPSC technology to model the hereditary cardiac arrhythmia syndrome. Both heterologous (HEK293/CHO-K1 cell expression systems) and iPSC-based cellular models are used to investigate the underlying disease mechanisms, and a comparison is conducted to evaluate whether the disease mechanisms are similar in both cell types. Results show differences on *I*_Ks_ channel function and trafficking in heterologous systems for five LQT1 patient mutations. The results are being modelled computationally to investigate the implications of *I*_Ks_ channel mutations on action potential duration and morphology, and the penetrance of the mutation in human cell populations such as those described in the previous session.

Dr Sara Dutta presented a state-of-the-art multiscale human whole heart computational framework to investigate arrhythmic effects of drugs and disease, and specifically hERG block in acute myocardial ischaemia. The framework presented here consists of a human anatomically based model with biophysically detailed representation of membrane kinetics including ionic current and concentration dynamics, as well as fibre orientation and ventricular heterogeneity. The human multiscale model is parameterized using experimental data, from voltage clamp at the ionic level to MRI scans at the whole heart level. They provide a multiscale platform to dissect and analyse specific ischaemic and arrhythmic processes with high spatiotemporal resolution, which is not possible to obtain through experiments alone, especially in human. The new insights provided by the human model could help in the design of further experimental and clinical studies to improve patient risk stratification, as well as decisions about drug dose during management of anti-arrhythmic therapy. This study^69^ can be extended to explore the mechanisms under different conditions, such as varying sizes and locations of the ischemic region, different drug compounds and their multichannel effects, and inter-subject variability in ionic currents and repolarization patterns. The presentation therefore highlighted the power of human multiscale simulations using anatomically based heart models to investigate safety and efficacy of pharmacological action in lethal disease conditions.

Using a different computational approach, Dr Julien Oster presented advances on the computational detection of arrhythmia episodes in ECGs. The development of mobile technologies (mobile phones, tablets, etc.) for health services (mHealth) is currently rapidly growing for two main reasons: (i) cost reduction and (ii) access to resource-scarce communities.^70^ The importance for the automatic or semi-automatic detection of arrhythmias on ECG recordings was highlighted in the presentation, given the simplicity of data acquisitions and therefore the multiplication of such data. Manual expert analysis would be more a burden for the clinicians than help for the diagnosis of cardiovascular diseases. Machine-learning approaches have been demonstrated to be a powerful tool for such an analysis for several applications, such as atrial fibrillation (AF) episodes or ventricular ectopic beat detection. Many Holter softwares already require electrophysiologists or laboratory technicians to annotate beats clustered together automatically. Llamedo and Martinez^71^ recently suggested a technique requesting expert labelling of the clusters, outputted based on both morphological- and rhythm-based features. Other techniques for rhythm classification or AF episodes detection, based uniquely on the heart rhythm, have also been presented, and are implemented in implantable devices.^72^

Dr Oster's presentation focused on two major arrhythmic types: AF and premature ventricular contraction (PVC). A machine-learning approach has recently been proposed, through the implementation of a Support Vector Machine^73^ on a mobile phone.^74^ PVC is another type of ventricular arrhythmia, identified as a predictor for mortality after myocardial ischaemia.^75^ The application of an ECG morphology model-based Bayesian filtering^76,77^ was shown to be effective for PVC detection.^78^

**Figure 5:**
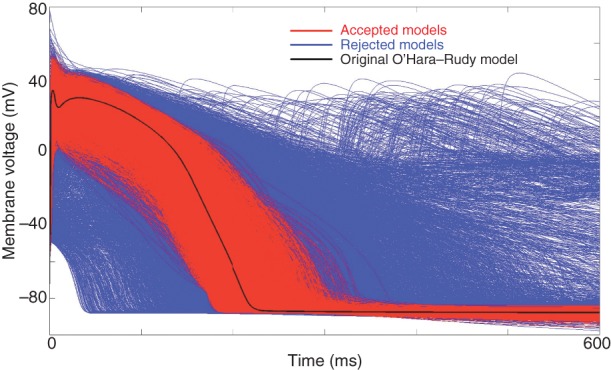
Population of human ventricular action potential models calibrated using *in vivo* electrogram recordings. Each simulated action potential in the population is generated using the O'Hara–Rudy model with ionic conductances sampled in a wide range of possible values. Calibration is then conducted using the *in vivo* electrograms by selecting the models that yield action potentials with properties such as action potential duration consistent with the electrograms (red traces, accepted models), and rejecting those that are outside range. In this figure, the action potential for the original O’Hara–Rudy model is shown in black.

Ms Xin Zhou presented computational investigations into the ionic mechanisms underlying repolarization alternans in a population of human ventricular models calibrated using *in vivo* recordings such as those presented by Prof. Taggart. Previous research into cardiac alternans has mainly been carried out in animals rather than in human. Ms Zhou presented the construction and calibration of a population of over 2000 human ventricular cell electrophysiology models to mimic the action potential variability exhibited in *in vivo* electrophysiological recordings from 41 patients.^79^ The *in silico* human ventricular cell population is illustrated in *Figure 5* and it was shown to reproduce two types of alternans restitution curves also observed in human *in vivo* recordings. By analysing the population of human *in silico* models, she dissected the mechanisms underlying cardiac alternans and how the complex interaction between sarcolemmal currents and calcium dynamics contribute to the initiation and maintenance of each alternans type. Therefore, in this presentation, multiscale human *in silico* models were used to capture key repolarization properties of *in vivo* recordings, to investigate the ionic mechanisms underlying the occurrence of proarrhythmic repolarization alternans and to identify potential anti-arrhythmic targets.

Dr Hua Rong Lu described a new, non-invasive and translational biomarker—the index of cardiac electrophysiological balance (iCEB, the ratio between QT and QRS)–in drug and ischaemia-induced cardiac arrhythmias and in genetic LQT syndrome and Brugada syndrome. Currently used biomarkers may not be adequate to detect all types of drug-induced cardiac arrhythmias. Furthermore, there is also a need for a new biomarker to detect cardiac risks in patients with gene-defects in the heart such as LQT syndrome and Brugada syndrome, in patients with heart failure and to detect cardiac risks in sportsmen and women. iCEB was successfully developed and introduced in 2013,^80^ it may detect potential risks for drug-induced cardiac arrhythmias beyond LQT and Torsade de Pointes. It may also a be better than currently used biomarkers derived from animal models, such as the isolated rabbit left-ventricular wedge model, because iCEB is also detecting additional drug-induced potential cardiac arrhythmias by slowing conduction and QT-shortening.^80^ iCEB was also found to be significantly increased in patients in genetic LQT syndrome and significantly decreased in patients with Brugada syndrome.

### New perspectives/beyond the human heart

Dr Jean-Pierre Valentin introduced the background of compound testing strategies in response to ICH guidelines and how the CiPA initiative^6,81–83^ will develop a new non-clinical paradigm for cardiac safety evaluation of new drugs by shifting the focus away from QT prolongation to an assessment of proarrhythmia to mitigate against the thorough QT study. The proposals would be to consider using ion channel effects in tandem with utilizing the emerging technology of *in silico* assessment and stem-cell-derived cardiomyocyte effects, but not at the expense of clinical ECGs or an understanding of pharmacokinetics/pharmacodynamics.^6^ The initiative aims to deliver an implementation of recommendations initially by mid-2016 onwards; however, this is dependent on community support in understanding more about which experimental inputs, which models and which levels of predictive capacity are required.^6^ Finally, Valentin reminded the audience of a need to keep QT in perspective and to consider cardiac effects beyond QT as it only accounts for 4% of cardiac safety-related drug attrition.^84,85^ Among the adverse events that are observed are arrhythmia, tachycardia and changes in blood pressure.^84,85^

Dr Andre Ghetti set out the problem facing the development of pharmaceutical drugs in the translation between *in vitro* and animal studies to the clinical setting and the critical need to have stronger models at the non-clinical phase for improving success and understanding of drug action. The approach that Dr Ghetti's company AnaBios will be taking is to use primary human tissue to derive cells that can be used more reliably in predicting later clinical effects than *in vitro* or animal studies can alone. This work is potentially to be used in parallel to the development of *in silico* approaches that are better informed (parameterized) by the data being generated from these isolated human primary cells. Taken together with a much improved characterization of donors, including medical history, allows us to consider the concept of incorporating *real-world*-type data into models.

Dr Rebecca Burton presented her recent work on developing a cell culture model of neurally mediated arrhythmogenesis and non-invasive optical imaging methods being pursued at Oxford.^86,87^ Biological models with varying degrees of complexity have been developed to shed light on re-entrant arrhythmias and cardiac monolayers are one of the simplest models. The next stages of this research is to pursue remote monitoring of *in vitro* cell cultures that would increase experimental access, reduce the need to sacrifice additional animals, and spur the adoption by other laboratories working in allied research areas. There has been a proliferation of remote access platforms that offer promising functionality. Remote access imaging offers advantages to both ‘wet-lab’ and ‘dry’ experimentation (computational and mathematical modelling), and there is a need for platforms which offer secure and reliable implementation.

## Key challenges moving forward

In order to succeed in a programme of research and implementation for drug discovery and testing that takes full advantage of the state of the art in cardiovascular science, the workshop discussions identified five key challenges to be met

1. Each of the human-based methodologies and techniques presented at the workshop has advantages and disadvantages, as each is able to provide specific forms of data and information, while also leaving some gaps and therefore having limitations. For example,
  - while *in vivo* human experiments are in many respects the best form of data, there are severe practical and ethical restrictions on acquiring this form of data;
  - *ex vivo* human data suffer from many similar practical and ethical restrictions, but also from the fact that the techniques (such as cell isolation and voltage clamp) used can sometimes bring about non-trivial effects that need to be compensated for interpreting results;
  - The utility of *in vitro* experiments on stem-cell-derived cardiomyocytes depends on the quality of the cell type produced by differentiation and subsequent maturation. Currently, this restricts their meaningful use to certain questions depending on the functional integrity of key components which may need to be proven rather than assumed to be intact.
2. Computational approaches have the potential to bridge these different methodologies, by extracting more value from the data acquired from each, filling in gaps, and facilitating comparison and complementarity between them. This can be achieved through computational approaches for data analysis (signal and image processing to automatically capture and quantify properties, through the use of mathematics and computer science) and through multiscale modelling, which allows for the exploration of mechanisms of disease at different scales, and through making predictions, in the form of new hypotheses for experimental approaches to investigate further.
  Computational modelling and simulation can meet their potential only insofar as they interact in meaningful ways with experimental methodologies and techniques. From an industry perspective, prediction would likely be predominantly used early in drug discovery for prioritization and aiding decision-making in compound selection. Later in drug discovery, the utility is likely to shift to providing a mechanistic understanding of *in vivo* results either to mitigate safety concerns or provide insights into mechanisms for efficacy in, e.g. antiarrhythmia indications. If computational approaches are developed in a close dialogue with experimental, clinical, and newly emerging digital techniques, they act as mediators between the different data forms and methodologies, and help to forge links between them.
3. The success of the integration of complementary forms of data and of methods depends upon achieving a true interdisciplinarity of approaches and people. There is a need for expertise from a wide spectrum of disciplines, together with the development of skills at reaching across disciplines and for communicating with researchers who are experts in different methods, and who have different perspectives and priorities. New ways of communicating are called for, as well as new approaches to training next-generation researchers who are capable of interdisciplinarity. In this respect, interdisciplinarity should be widened to include social studies of science in order to get a better grip on institutional, organizational, and social barriers.
4. Complementarity between different approaches requires input and investment from the scientific community, who are key to defining the criteria to be met for the assessment of drugs and models, through establishing benchmarks, as well as through a reconsideration of different methods and approaches to model validation. A consensus for how the pharma and biotech industry should respond is required and ideally supported by compelling data that draw on a retrospective analysis supporting the reasons to change. The motivation to change in pharma will be aided by the proposed reduced requirements for thorough QT studies and for the opportunity to reconsider compounds that previously have been discontinued due to, e.g. QT prolongation. Implementation will be somewhat dependent upon existing capabilities within the company, e.g. those capable of ion channel screening assays, stem cell assays, and *in silico* modelling. Even for those where this technology is established, the choice of protocols and standards is key, especially given the laboratory-to-laboratory variance in experimental measurements. From the modelling perspective, which model and which parameter set(s) need to be carefully defined; it would also be necessary to consider CRO partners for these organizations where ion channel screening, stem cell assays, and *in silico* modelling are impractical.
5. Finally, further measures to ensure a robust community formed around strong partnerships across different sectors need to be taken, including co-funding strategies that are targeted at developing the required research capability in people and organizations. Robust community formation requires attention to the social elements of the community as well as the scientific research questions.^88^ The further development of human-based methods have ethical as well as scientific advantages, since they aim to contribute to the 3Rs of animal experimentation; however, they are associated with other forms of social, ethical and legal constraints, and depend on successful engagement with the wider non-scientific community.

## Conclusion

In conclusion, the workshop identified key challenges in developing an integrative human-based approach to pharmacology and cardiology through the combination of *in silico, in vivo, ex vivo*, and *in vitro* approaches. A clear outcome of the discussions is that these challenges must be tackled synergistically, through joint efforts and discussions across the sectors and stakeholders. A creative strategy that is able to exploit complementarities between approaches needs to be designed and implemented, in a concerted community endeavour that is fully interdisciplinary and intersectoral.

## Funding

Financial support was provided by the Knowledge Exchange Fund of the University of Oxford, Wellcome Trust fellowships to B.R. (100246/Z/12/Z) and M.J.D. (WT098519MA), a British Heart Foundation Intermediate Basic Science Research Fellowship to S.H. (FS/12/59/29756), scholarships to S.D., L.C.N. O.J.B., and A.M. from the EPSRC, to A.L. from the British Heart Foundation Centre of Research Excellence, and to X.Z. from the China Scholarship Council. Funding to pay the Open Access publication charges for this article was provided by the Wellcome Trust.
